# A Pilot Study on Intravesical Administration of Curcumin for Cystitis Glandularis

**DOI:** 10.1155/2013/269745

**Published:** 2013-05-22

**Authors:** Qiong Lu, Fen Jiang, Ran Xu, Xiao-Kun Zhao, Zhao-Hui Zhong, Lei Zhang, Hong-Yi Jiang, Lu Yi, Yi Hou, Xuan Zhu

**Affiliations:** ^1^Department of Pharmacy, Second Xiangya Hospital of Central South University, Changsha 410011, China; ^2^Department of Pharmacology and Pharmacogenomics Research Center, Inje University College of Medicine, Busan 614-735, Republic of Korea; ^3^Department of Urology, Second Xiangya Hospital of Central South University, No. 139 Renminzhonglu-Road, Furong-District, Changsha 410011, China

## Abstract

Cystitis glandularis (CG) is a proliferative disorder in the urinary bladder. The outcome of current treatments in some patients is not satisfactory. Curcumin, a herbal medicine that has been used for centuries, has shown great potential in treating various diseases. Our pilot study aimed to explore the feasibility of an intravesical treatment for CG using curcumin. 14 patients diagnosed with CG that remained symptomatic after primary treatments were enrolled, underwent a 3-month curcumin intravesical treatment (50 mg/50 mL, 1 hour, once per week for first 4 weeks and once per month for next 2 months) and were followed up for 3 months. Efficacy of the treatment was evaluated using core lower urinary tract symptom score (CLSS) questionnaire. 10 patients demonstrated persistent improvement in symptoms up to the end of the 6-month study. Their CLSS decreased significantly after the 3-month treatment (6.0 ± 0.8; *P* < 0.01) from the baseline (10.5 ± 1.6) and maintained decreasing till the end of the study (6.2 ± 0.7; *P* < 0.01). 4 patients were classified as nonresponders. Our study suggests the feasibility of further randomized controlled trials on curcumin intravesical treatment in CG patients who remain symptomatic after primary treatments.

## 1. Introduction

Cystitis glandularis (CG) is a metaplastic alteration of the urothelium in the urinary bladder due to persistent infection, calculi, bladder exstrophy, outlet obstruction, or even tumor [1]. Morphologically, CG is subdivided into two subtypes: typical CG and intestinal CG [2]. It is classified as a benign disorder but there are also a few case reports of the conversion of CG into adenocarcinoma [3]. The typical symptoms of CG are urinary frequency, urgency, dysuria, and hematuria [4]. Clinically, the primary treatments for CG include cause removal, long-term antibiotic therapy, transurethral resection, and so forth [5, 6]. Outcomes of these treatments are sometimes unsatisfactory and some patients either remain symptomatic or experience recurrence afterwards. Nevertheless, appropriate adjuvant or subsequent therapy has not been developed with clinical evidence. Recently, intravesical chemotherapy has been suggested to treat CG, but the alternative drugs are limited [7].

Curcumin is the major component of turmeric, the powdered root of *Curcuma longa*, which is commonly used as a spice and food-coloring agent. It is known for its anti-inflammatory, antioxidant, and anticancer properties [8]. In recent years, oral curcumin, as a medicinal treatment, has gained popularity because of the successful experience in treating precancerous lesions of urinary bladder, oral leukoplakia, and intestinal metaplasia of the stomach [9–11]. More importantly, the safety, tolerability, and nontoxicity of curcumin at high doses are well established by human clinical trials [12].

Recently Hartojo et al. has discovered that curcumin may inhibit progression of Barrett's metaplasia [13], a disease that mimics CG etiologically and histologically [14]. In one animal study, curcumin was applied intravesically to treat bladder tumors [15]. We thus wondered if curcumin, when applied intravesically, would also be beneficial in patients with CG, and we designed a pilot study which aimed to evaluate the feasibility of an intravesical treatment with curcumin in CG patients who remained symptomatic or experienced recurrence after the primary treatments.

## 2. Materials and Methods

### 2.1. Participants

**Figure 1:**
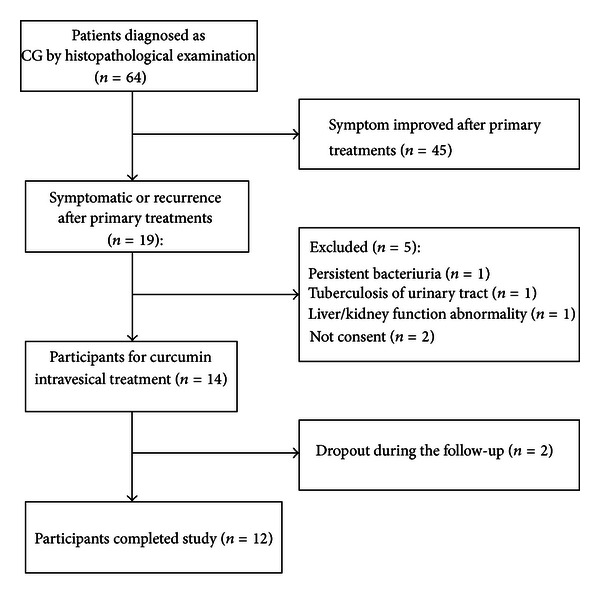
The CONSORT flowchart illustrating the recruitment of patients in the pilot study.

Among all outpatients admitted to the Department of Urology in Second Xiangya Hospital, Changsha, China, between Jan 2008 and Jan 2012, 64 patients were histopathologically diagnosed with CG. We aimed to recruit the CG patients who remained symptomatic after at least 3-month primary treatments; but those who had persistent bacteriuria after primary treatments, tuberculosis of urinary tract, and liver or kidney function abnormality were excluded. The CONSORT flowchart of the study is presented in Figure 1. In the end, 14 patients were included in the study and the demographic information is described in Table 1. All patients ceased antibiotic therapy more than 3 days prior to the clinical trial. The study protocol was approved by the Institutional Review Board of the Second Xiangya Hospital, Changsha, China, and written informed consent was obtained from each patient.

### 2.2. Intravesical Curcumin Administration

The curcumin powder (curcumin ≥ 80%; product number C7727) was purchased from Sigma-Aldrich Co (St. Louis, MO, USA). The dosage we used referred to a previous study of curcumin intravesical treatment in mice [15]. Following bladder catheterization, the patients received 50 mg curcumin dissolved to 50 mL 5% sodium bicarbonate and restrained for 1 hour. The treatments were applied once per week for the first 4 weeks and once per month for the next 2 months, and the participants were then followedup for another 3 months. 

### 2.3. Evaluation of the Treatment

All patients were instructed to keep a 24-hour voiding diary and efficacy of the treatment was assessed using the core lower urinary tract symptom score (CLSS) questionnaire (see Supplemental Table 1 available at http://dx.doi.org/10.1155/2013/269745) a reliable tool in the overall assessment of lower urinary tract symptoms in male and female [16], in addition to routine urinalysis, before and 24 hours after the 4-week, the 3 months treatment, and at 6 months of the study. The responders accepted further cystoscopic examination. 

### 2.4. Statistical Analysis

Statistical analysis was performed with software SPSS 19.0 (SPS Inc., Chicago, IL, USA) using the nonparametric Wilcoxon Signed-Rank test to compare the CLSS after 4-week and 3-moth treatments with that from the baseline. *P* < 0.05 was considered statistically significant.

## 3. Results

**Figure 2:**
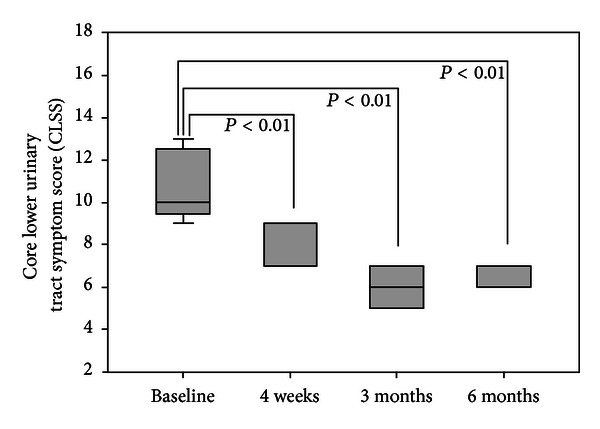
Box plots of the core lower urinary tract symptom scores (CLSS) from 10 responders at baseline, 4 weeks (24 hours after the 4-week weekly curcumin treatment), 3 months (24 hours after the 2-month monthly treatment), and 6 months (at the end of the 3-month follow-up). The top whisker (vertical line) = 95th percentile, top of box = 75th percentile, median = wide horizontal line, bottom of box = 25th percentile, and bottom whisker = 5th percentile. *P* < 0.01, compared with baseline.

All 14 patients showed good tolerance to the curcumin treatment and 12 of them completed the study (2 were lost to follow-up after the completion of the 3-month treatment). No obvious adverse events were observed. At the end of the 3-month curcumin treatment, 10 (71.4%) patients demonstrated a significant improvement in symptoms compared with that at the baseline; the CLSS of the 10 responders decreased from 10.5 ± 1.6 at baseline to 7.6 ± 1.1 after the 4-week treatment (*P* < 0.01), further decreased to 6.0 ± 0.8 after the 3-month treatment (*P* < 0.01), and was maintained at 6.2 ± 0.7 after the 3-month followup (*P* < 0.01) (Figure 2), the three most improved symptoms were day frequency, urgency, and straining (Supplemental Figure 1). Hematuria improved in 3 patients and disappeared in another 2 patients (Table 1). In addition, cystoscopic examination demonstrated improvement to various degrees in all responders; a significant improvement seen in patient 6 (Supplemental Figure 2). 4 (28.6%) patients were classified as nonresponders because their CLSS did not improve and 3 of them were identified as intestinal CG, and the other 1 was diagnosed with overactive bladder during the study. 

## 4. Discussion

Owing to the improved understanding toward molecular mechanisms of herbal medicines, more and more clinical studies have been conducted to either confirm the benefits of their traditional usage or to explore their new applications, such as of our current study. Since curcumin has rarely been used to treat urological diseases including CG in human patients, it is necessary to conduct a pilot study first to testify the feasibility of a randomized controlled clinical trial on intravesical administration of curcumin although there have been abundant mechanism studies which suggested its clinical application.

Generally, curcumin's nonspecific antioxidant, anti-inflammatory, and healing properties may contribute to the improvement in symptoms, in view of the benign proliferative nature of CG [12]. As to the uroprotective effect of curcumin, because it has been well noted that primary urothelial lining defects play an important role in chronic cystitis and bladder oversensitivity [17], curcumin may improve the energy status and restore the oxidant/antioxidant balance in urothelium through modulating the release of inflammatory endocoids, namely, TNF*α* and NO, which has been proved by Arafa using the cyclophosphamide haemorrhagic cystitis model [18]. In addition, high-level membranous expression of E-cadherin, *β*-catenin, and TNF*α* and increased telomerase activity may be involved in the pathogenesis of CG [14, 19]. Previous studies on curcumin showed that it significantly suppressed *TNF*α** gene products [20], attenuated *β*-catenin signaling through the activation of protein kinase D1 [21], also inhibited telomerase activity [22]. 

Oncogenically, although both types of CG may be precursors of adenocarcinoma [23], an intravesical chemotherapy or operation seems unnecessary since such cases are rare. However, curcumin has been used to treat various cancers and precancerous lesions [9–11] and also recognized as a promising antibladder cancer drug *in vitro* and *in vivo *[15]. Unlike chemotherapeutic drugs which often have serious side effects, curcumin is superior for its good safety and rare side effects [12].

In our study, 2 patients were lost to follow-up and the reasons were unknown; 4 (28.6%) patients were classified as nonresponders; 3 of them were diagnosed with intestinal CG with obvious causes unknown, suggesting a lack of efficacy of current curcumin treatment in cases of complicated etiology; the other 1 case was diagnosed with overactive bladder, a neuromuscular disease that we also have limited understanding to its etiology; moreover a previous study suggests that curcumin may increase muscle tone in urinary bladder [24], and thus the efficacy of curcumin may be limited in patients with overactive bladder. Since our study is preliminary, in order to confirm the beneficial effect of intravesical treatment of curcumin, randomized controlled clinical trials with different dosage and treatment period regimens are suggested. 

In this pilot study which included 14 CG patients, a 3-month curcumin intravesical therapy was well tolerated and resulted in significant improvement in symptoms for up to 6 months in 10 patients. Our pilot study clearly suggests the feasibility of further randomized controlled trials, with modified protocol on intravesical curcumin administration in CG patients who are resistant to conventional therapies.

## Supplementary Material

In CLSS questionnaire, ten lower urinary tract symptoms (LUTS) (increased daytime frequency, nocturia, urgency, urgency incontinence, stress incontinence, slow urinary stream, straining, feeling of incomplete emptying, bladder pain, and urethral pain) were selected as core LUTS. The CLSS questionnaire provides overall assessment of relevant symptoms without omissions and is useful for new patients, patients with multiple diseases, and patients without a definite diagnosis, as well as before and after interventions that may cause other symptoms.

## Figures and Tables

**Table 1:** Characteristics of curcumin-treated patients.

Patient	Sex	Age (years)	Subtype	CLSS				Hematuria			
				Baseline	4 weeks	3 months	6 months	Baseline	4 weeks	3 months	6 months
1	M	36	CGTP	10	7	5	6	Gross	Micro	Micro	Micro
2	M	58	CGIT	10	8	6	6	Gross	Gross	−	−
3	M	43	CGTP	9	7	5	5	Micro	Micro	Micro	Micro
4	F	64	CGIT	10	10	10	10	Micro	Micro	Micro	Micro
5	M	56	CGTP	13	9	7	6	−	−	−	−
6	F	30	CGTP	13	9	7	7	Micro	Micro	Micro	Micro
7	F	48	CGTP	10	7	6	7	Micro	Micro	Micro	Micro
8	M	23	CGIT	12	9	7	7	Micro	Micro	−	−
9	M	47	CGIT	10	11	10	10	Micro	Micro	Micro	Micro
10	F	42	CGTP	10	7	5	6	−	−	−	−
11	M	72	CGTP	10	10	10	ND	Micro	Micro	Micro	ND
12	F	18	CGTP	9	7	6	ND	−	−	−	ND
13	M	52	CGIT	9	9	9	9	Gross	Gross	Micro	Micro
14	M	32	CGTP	9	6	6	6	Gross	Micro	Micro	−

F: female; M: male; CGTP: typical CG; CGIT: intestinal CG; CLSS: core lower urinary tract symptom score; Micro: microscopic hematuria; Gross: gross hematuria; −: negative hematuria; ND: not determined due to loss to follow-up.
