# Supplementary material for: A Pilot Study on Intravesical Administration of Curcumin for Cystitis Glandularis
Source: Evid Based Complement Alternat Med. 2013 May 22;2013:269745. doi: 10.1155/2013/269745 (PMC3674727; doi:10.1155/2013/269745)
Supplement: Supplementary file 1 — In CLSS questionnaire, ten lower urinary tract symptoms (LUTS) (increased daytime frequency, nocturia, urgency, urgency incontinence, stress incontinence, slow urinary stream, straining, feeling of incomplete emptying, bladder pain, and urethral pain) were selected as core LUTS. The CLSS questionnaire provides overall assessment of relevant symptoms without omissions and is useful for new patients, patients with multiple diseases, and patients without a definite diagnosis, as well as before and after interventions that may cause other symptoms. [file 269745.f1.doc]

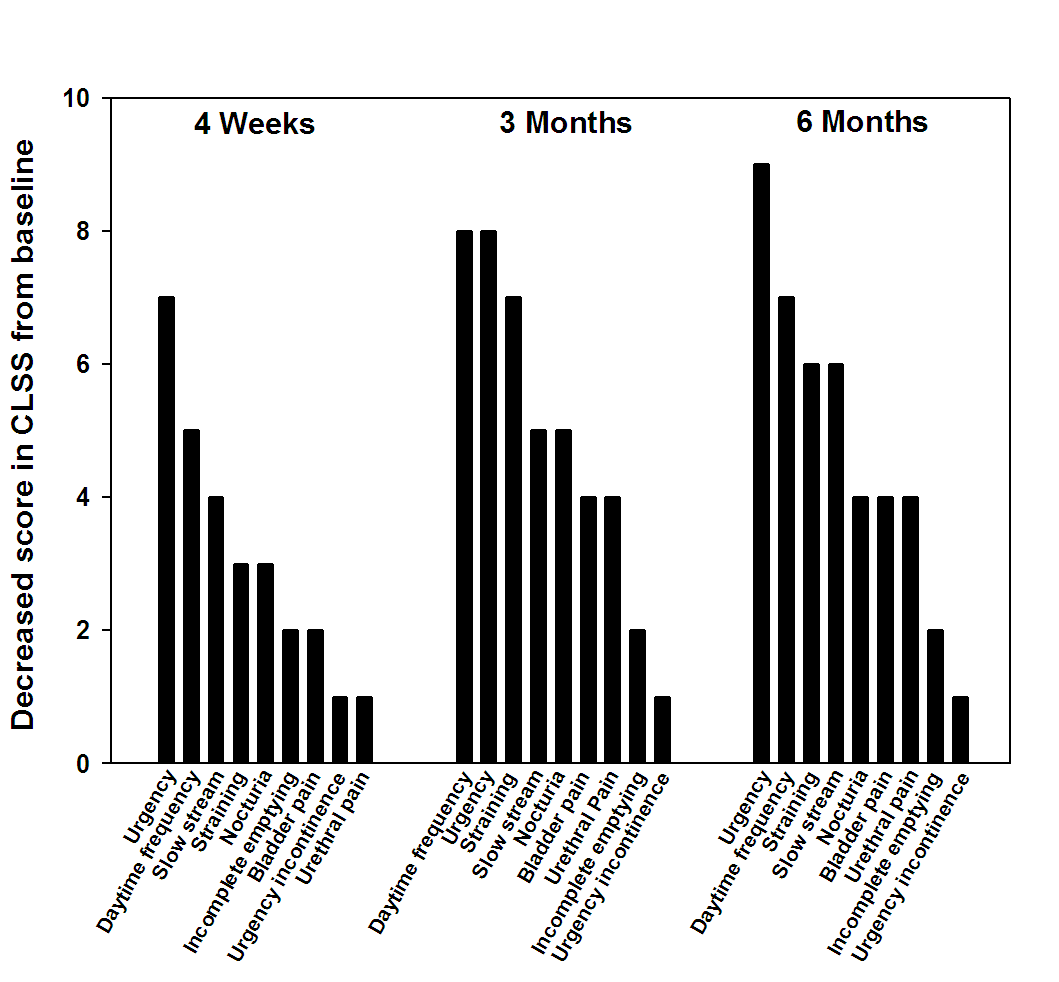


**Supplemental Figure 1** Sum of the decreased scores in each question in CLSS from the baseline in 10 responders.


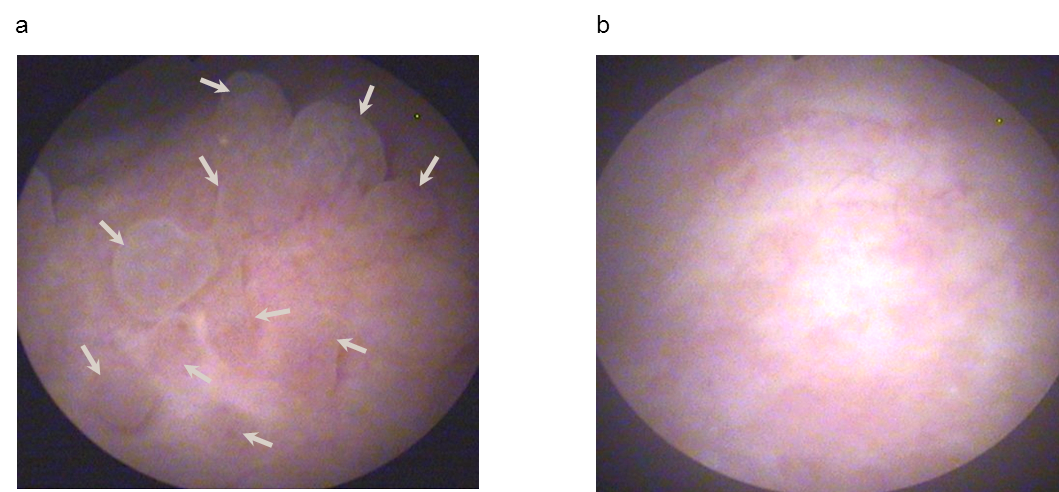


**Supplemental Figure 2** Cystoscopy images before (a) and after (b) curcumin intravesical treatment in a 30 year-old female patient diagnosed with typical cystitis glandularis, featured by a cobblestone appearance of the mucosa with focal polypoid masses (arrows) in (a) and a disappearance of the lesions in (b).

**Supplemental Table 1** Core Lower urinary tract Symptom Score (CLSS) questionnaire

| 1. How many times do you typically urinate from waking in the morning until sleeping at night? | 0 | 1 | 2 | 3 |
| --- | --- | --- | --- | --- |
|  | -7 | 8-9 | 10-14 | 15- |
| 1. How many times do you typically urinate from sleeping at night until waking in the morning? | 0 | 1 | 2 | 3 |
|  | 0 | 1 | 2-3 | 4- |

| How often do you have the following symptoms? | no | rarely | sometimes | often |
| --- | --- | --- | --- | --- |
| 1. A sudden strong desire to urinate, which is difficult to postpone | 0 | 1 | 2 | 3 |
| 1. Leaking of urine because you cannot hold it | 0 | 1 | 2 | 3 |
| 1. Leaking of urine, when you cough, sneeze, or strain | 0 | 1 | 2 | 3 |
| 1. Slow urinary stream | 0 | 1 | 2 | 3 |
| 1. Need to strain when urinating | 0 | 1 | 2 | 3 |
| 1. Feeling of incomplete emptying of the bladder after urination | 0 | 1 | 2 | 3 |
| 1. Pain in the bladder | 0 | 1 | 2 | 3 |
| 1. Pain in the urethra | 0 | 1 | 2 | 3 |
|  | CLSS (Sum of Q1-10) ______ | | | |
